# Editorial: Biodegradation of plastics

**DOI:** 10.3389/fbioe.2023.1150078

**Published:** 2023-04-03

**Authors:** Martin Koller, Ken’ichiro Matsumoto, Zhanyong Wang, Fan Li, Aamer Ali Shah

**Affiliations:** ^1^ Research Management and Service, Institute of Chemistry, University of Graz, Graz, Austria; ^2^ Research Group of Biotechnology, Laboratory of Biomolecular Engineering, Faculty of Engineering, Division of Applied Chemistry, Hokkaido University, Sapporo, Japan; ^3^ Biological Science and Technology College, Shenyang Agricultural University, Shenyang, China; ^4^ School of Life Sciences, Northeast Normal University, Changchun, China; ^5^ Department of Microbiology, Quaid-i-Azam University, Islamabad, Pakistan

**Keywords:** biodegradability, plastic, plastic wastes, microbial biodegradation, enzymes

Currently, humankind faces severe threats connected to aggravating climate crisis, shortage of fossil resources, and increasing quantities of waste consisting of petrochemistry-based plastics. Despite the fact that such plastics, since the mid of the last century, brought numerous benefits and advantages to our society by being stable, versatile, and of low specific mass, they have a dark side: Due to the lacking adaptation of nature to these xenobiotic materials, which were not present on Earth only some decades ago, they are highly recalcitrant towards biodegradation, i.e., their disintegration into non-toxic final products such as CO_2_ or CH_4_ by the action of living organisms or biocatalytic parts thereof. One could literally state that “plastics are forever!” Nowadays, reports estimate a total quantity of almost three billion tons having been produced since the onset of plastic commercialization; about 400 million tons plastics are being produced annually at the moment, with a strongly increasing trend especially in emerging and developing countries (Mukherjee and Koller, 2022). Only part of spent plastic waste undergoes a controlled end-of-life scenario like mechanical or chemical recycling or thermal conversion for energy recovery, while this later option is heavily disputed due to the obstacles connected to formation of surplus CO_2_ and toxic gases. Moreover, existing recycling and incineration plants are of insufficient capacities to handle the steadily increasing quantities of plastic waste of petrochemical origin, especially full-carbon-backbone plastics like polyolefins, but also poly (ethylene terephthalate) (PET) or poly (urethanes). However, the lion’s share of spent plastic is littering aquatic and terrestrial environments as macro- and microplastic waste, causing impacts on the biosphere that are only just beginning to be fully understood.

Currently, there are two scenarios to overcome these issues: One option is to switch to plastic biopolymers. Tremendous research is currently dedicated to such materials, which are both biobased and biodegradable, with microbial polyhydroxyalkanoates (PHA) probably being the most prominent example. However, there are still impediments on the way towards replacing petroplastics by bio-inspired alternatives, such as high production costs, fluctuating material quality, and an often-lacking match between customer expectations and *de facto* material performance. The second option is biodegradation of present petroplastics, as it is the topic of the Research Topic at hand.

During the last years, several organisms have been shown to contribute to degradation of said petrochemical plastics. However, the number of these organisms is still scarce, and these processes occur at rather low rates. The enzymatic machinery responsible for plastic biodegradation typically shows low affinity to the plastic substrate, operates with insufficiently deciphered reaction mechanisms, or is inefficiently expressed by the organisms. Especially, we are aware of a deep knowledge gap between preliminary research data, postulating degradability of a given polymer by certain strains or enzymes on lab scale, and, on the other hand, large scale degradation experiments performed under diverse realistic environmental conditions. What is needed to overcome existing bottle necks are progress in the field of microbiology of plastics’ biodegradation and advanced biotechnological approaches. New powerful strains are waiting for their discovery as toolboxes containing enzymes for plastic biodegradation. This research needs to encompass the holistic elucidation of the metabolic pathways active during plastic biodegradation, identification of relevant genes and enzymes expressed by them, assessment of enzymes in terms of activity and stability, and genetic engineering to obtain tailor-made whole cell biocatalysts for plastic biodegradation, and strategies of biosynthetic biology, supported by bioinformatic tools.

So, there is definitely a lot to be achieved in this challenging scientific field, and a lot of research activity in this direction is witnessed globally. This gave reason to launch this Research Topic of Frontiers in Bioengineering and Biotechnology dedicated to Biodegradation of Plastics. A total of nine research groups of exceptional reputation in the global scientific community contributed their recent research outcomes to this issue, which contains eight original research articles and one comprehensive review paper. The subsequent paragraphs present the individual specific contributions in a nutshell:

In their comprehensive review, Urbanek et al. introduce the current state of research on biodegradation of PET, a heavily utilized petroplastic polymer, which frequently undergoes mechanical recycling, thus contributing to microplastic formation. Authors demonstrate that isolated wild type organisms showing PET degrading activity are of insufficient efficiency to provide a solution to overcome the problems associated to PET waste. Therefore, it is shown how strategies of metabolic engineering of microbes and protein engineering can enhance biodegradation of PET.

Jiang et al. dedicated their article to biodegradation of the polyolefin poly (styrene) (PS), a full-carbon-backbone petroplastic also produced at enormous quantities. Authors were able to isolate a PS-degrading bacterium from the digestive system of *Galleria mellonella* wax moth larvae that were fed with PS foam. Isolated bacterium was identified as *Massilia* sp. FS 1903. Biodegradation of PS films by this bacterium was studied *via* scanning electron microscopy and X-ray energy dispersive spectrometry, and water contact angle measurements quantified the increasing hydrophilicity of PS films during incubating with the bacterium by formation of oxygen-bearing groups on the surface. Incubation experiments demonstrated a significant mass loss of PS films after 30 days, which outperforms results for PS biodegradation reported for other biological systems.

Poma et al. resorted also to *G. mellonella* wax moths in the larval stage for plastic degradation studies. Here, authors aimed at biodegradation of low-density poly (ethylene) (LDPE). Insects were conditioned by three different beekeeping residues (beeswax, balanced diet, and wheat bran), and fed with LDPE for different times. LDPE biodegradation was monitored *via* RAMAN spectroscopy and mass loss measurements. Best results for LDPE biodegradation were obtained when exposing larvae conditioned with beeswax for 36 h.

Dar et al. addressed the topic of “plasticulture,” hence, the agricultural application of plastics. These authors developed and characterized novel potentially biodegradable polymers. On the one hand, polymers consisting of ionic liquid monomers were prepared *via* photo radical induced polymerization; on the other hand, PE-like n-alkane disulfide polymers were produced from 1,ω-di-thiols through thermally activated air oxidation. These polymers were subjected towards soil biodegradation studies under controlled conditions, monitoring the formation of volatile degradation products by a respirometer and an advanced proton-transfer-reaction time-of-flight mass spectrometer system. While polymers based on ionic liquids did not demonstrate significant biodegradation, the disulfide-based polymer of 1,10-n-decane dithiol reached 20% degradation under basic soil conditions at room temperature within 3 months. This study demonstrates that introducing disulfide groups into the PE backbone is an option to make this material higher prone to biodegradation.

Li et al. studied the performance of two extracellular enzymes produced by *Pseudomonas hydrolytica* sp. DSWY01T for biodegradation of the synthetic polyester poly (ε-caprolactone) (PCL). Enzymes were purified, characterized, and the related genes were analyzed in details. Two novel PCL-degrading enzymes, PCLase I and PCLase II, were studied; both enzymes were able to hydrolyze PCL into monomers and oligomers, with PCLase I having shown to outperform biodegradation performance of PCLase II. *Via* sequence analysis and substrate specificity analysis, it was shown that PCLase I and PCLase II were a cutinase and a lipase, respectively, with optima for temperature and pH-value at 50°C and 9.0 (PCLase I) and 40°C and 10.0 (PCLase II).

A related organism, *Pseudomonas chlororaphis* PA23, was considered by Mohanan et al. for enzymes catalyzing biodegradation of different (bio)polymers. Two genes encoding the intracellular lipases LIP1 and LIP2 were identified, characterized and expressed in the host *Escherichia coli*. Encoded lipases LIP1 and LIP2 revealed strongly different amino acid sequences, catalytic mechanisms, substrate specificities, and polymer hydrolysis rates. It was shown that the recombinant LIP1 expressed in *E. coli* had best hydrolytic activity at 45°C and pH 9.0, this activity increased in presence of calcium cations. Maximum activity of LIP2, which was unaffected by calcium, was demonstrated at 40°C and pH 8.0. Both enzymes exhibited a broad substrate range; LIP1 and LIP2 were able to hydrolyze different types of PHAs, polylactic acid (PLA), and para-nitrophenyl (pNP) alkanoates. Moreover, some hydrolytic activity of these enzymes on the synthetic plastics PCL and poly (ethylene succinate) (PES) was discovered, thus making them of interest for biodegradation of polyesters of petrochemical origin.

In their study, Li et al. developed poly (hexamethylene succinate-*co*-ethylene succinate) [P(HS-*co*-ES)] copolymers with varying physical properties and enzymatic hydrolyzability *via* melting polycondensation of monomers (hexylene succinate and ethylene succinate). Properties (crystallinity, thermal and mechanical characteristics, wettability, and susceptibility to enzymatic attack) were varied by changing the ratio of the two monomers. Enzymatic hydrolysis rates of all prepared copolyesters outperformed those of the corresponding homopolyesters [poly (hexylene succinate) and poly (ethylene succinate)]. It was shown that the copolyester containing 51% ethylene succinate did not fully degrade; this material is of interest for long-term applications. In contrats, copolyesters with 13% and 76% ethylene succinate rapidly degraded; they are auspicious materials for short-term applications, where fast degradation is desired.

Andler et al. addressed the problems associated to the enormous quantities of car tire waste consisting of vulcanized rubber, which is very resistant towards biodegradation due to its highly hydrophobic cross-linked structure, very hard to recycle, and contributes significantly to the global microplastic formation. Authors investigated a total of ten fungal strains over a period of 1 month for their biodegradation performance of vulcanized rubber particles; mass loss and surface structure changes were monitored. As major outcome, cultivations of the white rot fungi *Trametes versicolor* and *Pleurotus ostreatus* achieved a mass reduction of the polymer of 7.5% and 6.1%, respectively, after 4 weeks. Genome sequence analysis of both strains showed that these organisms possess a higher number of sequences for laccases and manganese peroxidases, two crucial extracellular enzymes for many oxidative reactions. This was experimentally confirmed by the high activity of laccase and peroxidase when cultivating the two fungal strains on rubber particles in comparison to rubber-free cultivation setups. Authors suggest that their results might pave the way towards efficient bio-inspired solutions for vulcanized rubber biodegradation in the future.

An intriguing approach for combining plastic upcycling and production of biopolymers was presented by Esmail et al. These researchers found out that the bacterial strains *Komagataeibacter xylinus* DSM 2004 and DSM 46604 are able to produce bacterial cellulose, a polysaccharide of high market potential, from conversion of terephthalic acid and ethylene glycol, the building blocks of the petroplastic PET. The two strains revealed different cultivation performance on these unusual substrates; strain DSM 2004 achieved higher productivity for bacterial cellulose than strain DSM 46604, which, in contrast to DSM 2004, was unable to grow on ethylene glycol as the sole carbon source, but utilized mixtures of ethylene glycol and terephtalic acid. For both strains, cellulose formation was enhanced by co-feeding of glucose. A new downstream process was developed in this study for preparation of highly pure bacterial cellulose, encompassing dissolution of the polysaccharide in aqueous NaOH solution. Interestingly, bacterial cellulose produced by *K. xylinus* DSM 2004 and DSM 46604, respectively, showed different crystallinity and fiber diameters.

We are convinced that respected readers will draw the necessary inspiration from the presented articles for their own research work in the field of plastic biodegradation and related scientific areas. Most of all, we expect we expect that this book will provide the scientific community with the brainstorm it needs to eventually come up with efficient solutions to deal with the plastic dilemma.
